# Complete, circular genome sequence of a *Bosea* sp*.* isolate from soil

**DOI:** 10.1128/MRA.00360-23

**Published:** 2023-08-21

**Authors:** Maria Alvarez-Arevalo, Eva Baggesgaard Sterndorff, David Faurdal, Anna-Sophie Mourched, Pep Charusanti, Tue Sparholt Jørgensen, Tilmann Weber

**Affiliations:** 1 The Novo Nordisk Foundation Center for Biosustainability, Technical University of Denmark, Kongens Lyngby, Denmark; University of Southern California, Los Angeles, California, USA

**Keywords:** *Bosea*, rare soil microbes, Alphaproteobacteria, nanopore

## Abstract

Here, we report the complete, circular genome sequence of a potential novel species from the underexplored Alphaproteobacterial genus *Bosea. Bosea* sp. NBC_00550 was isolated from a soil sample collected in Lyngby, Denmark. We explore the biosynthetic potential of *Bosea* sp. NBC_00550 and compare it with that of other *Bosea* species.

## ANNOUNCEMENT

Among the Alphaproteobacteria living in soils, *Bosea* is underexplored, and at the time of writing, only seven complete genomes had been deposited at NCBI. In this paper, a strain divergent from previously sequenced isolates is presented to increase diversity to the genome database of this genus.

*Bosea* sp. NBC_00550 was isolated from a soil sample collected in Lyngby, Denmark (SAMN29888037, 55.798056 N 12.541667 E) at 3–5 cm depth as described in reference (1). The strain was isolated by plating on solid ISP4 medium and incubating at 30°C (2). A single colony was picked, grown in liquid medium, and frozen in 25% vol/vol glycerol. The frozen isolate was inoculated directly in liquid ISP2 medium and incubated for 5 days at 30°C, 140 rpm before DNA extraction using a modified QIAGEN Genomic-tip 20 protocol according to references (3, 4). Default parameters were used for all software and protocols unless otherwise specified.

A NEBNext Ultra II DNA library with six PCR cycles yielded 10,661,381 read pairs from an Illumina NovaSeq machine using a 2 × 150 bp strategy. Illumina reads were trimmed using Trim Galore (5) [v. 0.6.4_dev, running Cutadapt v. 2.10 (6)] with a minimum length of 100 bp and minimum quality of Q20.

A Nanopore library of *Bosea* sp. NBC_00550 was constructed using DNA from the same extraction as was used to generate the Illumina data. A MinION 9.4.1 flowcell and the SQK-RBK110.96 kit were used to generate Nanopore data, which were basecalled using the high-accuracy model in Guppy (v. 5.0.17 + 99baa5b). Barcodes were trimmed in the MinKNOW software. As a result, 1,164,240,587 bp with an N_50_ of 10,514 bp was used for assembly.

A Flye assembly (v.2.9-b1768, --iterations 5) (7) was polished with Illumina data using Polypolish (v.0.5.0) (8) and POLCA (v.4.0.5) (9) . The genome integrity was evaluated with Bandage (v.0.8.1) (10), and the core gene content was evaluated with BUSCO (v.5.1.2, database alphaproteobacteria_odb10) (11). The low number of bases in the assembly not covered by the Illumina reads indicates a very high assembly quality (Table 1, reported by Polypolish). The taxonomy was determined with GTDBtk (v.1.7.0) (12), and NCBI PGAP (v.6.3) (13) was used for automated annotation. AntiSMASH (v. 6.1.1) (14) was used to identify biosynthetic gene clusters (BGCs), and Clinker (v.0.0.24) (15) was used to compare the gene clusters to *Bosea* complete genomes from the NCBI database.

**TABLE 1:** Accession numbers and assembly characteristics of the sequenced *Bosea* strain

		*Bosea* sp. NBC_00550	
Assembly length (bp)		6,267,852	
Total BUSCO groups searched: 432	Complete and single copy	430	
	Complete and duplicated	2	
	Fragmented	0	
	Missing	0	
PGAP^*a*^ annotation	No. of CDS^*b*^	5,908	
	No. of tRNAs	50	
	No. of rRNAs	2, 2, 2 (5S, 16S, 23S)	
Detected BGCs by antiSMASH (14)		Three regions: terpene, NAPAA, NRPS-T1PKS hybrid	
BioProject		PRJNA861150	
BioSample		SAMN29888037	
Assembly accession number		GCA_026020075.1	
GenBank accession no.		CP102772	CP102773
Locus name		NBC_00550 chromosome	Plasmid pNBC550
Topology		Circular	Circular
Length (bp)		5,687,190	580,662
Nanopore coverage (×)		194	116
Illumina coverage (×)		500.1	372.5
Uncovered bases (bp)		2	2
Closest GTDBtk hit (genus, species, accession number, %ANI)		*Bosea sp.* F3-2, GCF_008253865.1 88.05 %ANI	
Complete *Bosea* genomes from NCBI (accession numbers)	GCA_001741865, GCA_008253865, GCA_003952665, GCA_002220095, GCA_001562255, GCA_011764485, GCA_001713455		

^*a*^ PGAP, Prokaryotic Genome Annotation Pipeline.

^*b*^ CDS, coding sequences.

The assembly produced a circular chromosome of 5.7 Mbp with 66% GC and a large circular plasmid of 581 kbp with 63% GC (Table 1). The Nanopore and Illumina coverages were 194 and 500, respectively, and 432 (100%) BUSCO genes were complete. *Bosea* sp. NBC_00550 carries a non-alpha poly-amino acid (NAPAA) BGC with the specificity-conferring code DLedLgTVvK, which is also found in all seven other complete *Bosea* genomes (Table 1), and a terpene cluster is also found in all other seven *Bosea* genomes. The complete *Bosea* genomes carry between three and nine regions encoding BGCs, and in addition to the shared NAPAA and terpene BGCs, *Bosea* sp. NBC_00550 carries a hybrid NRPS-T1PKS cluster, which is similar to a hybrid NRPS-T1PKS BGC found in *Bosea vaviloviae* (GCA_001741865). AntiSMASH predicts that the NRPS-T1PKS hybrid has glycosyltransferases and polysaccharide biosynthesis genes near the small core NRPS-T1PKS gene.

## Data Availability

Data are available as BioProject PRJNA861150. SRA accession numbers for raw reads are SRR20852345 and SRR20852344. GenBank accession numbers are listed in Table 1.
